# Equation Predicts Renal Function after Nephroureterectomy to Treat Upper Tract Urothelial Carcinoma

**DOI:** 10.1590/S1677-5538.IBJU.2025.0076

**Published:** 2025-09-30

**Authors:** Kai-Sen Su, Hsiao-Jen Chung, Yen-Hwa Chang, William J.S. Huang, Eric Yi-Hsiu Huang

**Affiliations:** 1 Taipei Veterans General Hospital Department of Urology Taipei Taiwan Department of Urology, Taipei Veterans General Hospital, Taipei, Taiwan; 2 National Yang Ming Chiao Tung University School of Medicine and Shu-Tien Urological Science Research Center Department of Urology Taipei Taiwan Department of Urology, School of Medicine and Shu-Tien Urological Science Research Center, National Yang Ming Chiao Tung University, Taipei, Taiwan

**Keywords:** Carcinoma, Transitional Cell, Kidney function test, Renal plasma flow, Urologic neoplasm

## Abstract

**Purpose:**

A preoperative tool is warranted to predict renal function after radical nephroureterectomy, as this would optimize treatment plans and avoid postoperative end-stage renal disease. Therefore, this study aimed to develop an equation to predict postoperative renal function changes.

**Materials and methods:**

We reviewed the medical records of 487 patients who were diagnosed with upper tract urothelial carcinoma treated by unilateral radical nephroureterectomy with bladder cuff excision between 2010 and 2020. Renal function was comprehensively evaluated preoperatively using 99mTc-mercaptoacetyltriglycine renal scintigraphy. Serum creatinine was evaluated before, and 3 and 6 months postoperatively. We then developed a predictive equation using Pearson linear correlation analysis.

**Results:**

The median preoperative effective renal plasma flow was 241.30 mL/min., of which the lesion side accounted for 35.6% of the total. A predictive equation for changes in postoperative renal function was established. The preoperative lesion-side effective renal plasma flow ratio was significantly associated with an estimated glomerular filtration rate (eGFR) decline ratio at 3 months postoperatively (r = 0.613, P < 0.001). Internal validation of the patients during 2021 confirmed that the equation could predict eGFR decline (r = 0.896) and radical nephroureterectomy-related cisplatin ineligibility (100%) at 3 months postoperatively.

**Conclusions:**

We established an equation that assists in predicting changes in postoperative eGFRs based on the preoperative lesion-side effective renal plasma flow ratio. This equation can also predict postoperative residual renal function.

## INTRODUCTION

Upper urinary tract urothelial carcinoma (UTUC) is a rare malignancy that accounts for 5%–10% of urothelial cancers ( 1 ). Radical nephroureterectomy with bladder cuff excision (RNU-BCE) is the standard treatment for high-risk nonmetastatic UTUC. The contribution of the remaining contralateral kidney to postoperative residual renal function is a major issue that could substantially affect preoperative decision-making. A preoperative tool is warranted to predict renal function after RNU.

Serum creatinine and estimated glomerular filtration rates (eGFRs) are the simplest and most popular methods for evaluating renal function in clinical practice. Impaired renal function post-RNU has been predicted on the basis of eGFRs ( 2 - 4 ). Both kidneys impact overall function, so split function must be assessed to predict contralateral performance post-surgery.

The continuous development of nuclear medicine has rendered split renal function tests before unilateral nephrectomy accessible and indispensable, especially when postoperative residual renal function is a concern. Effective renal plasma flow (ERPF) measured using angiographic radiotracer perfusion, is a prevalent split renal function test that includes plasma sampling and renography. While previous studies have explored renal function prediction using nuclear medicine, a reliable scintigraphy-based model tailored to UTUC patients, with potential relevance to perioperative systemic therapy, has yet to be developed ( 5 - 8 ). Our objective is to create a predictive equation that utilizes preoperative ERPF as a single factor to estimate postoperative eGFR in patients with UTUC undergoing unilateral RNU, with the hypothesis that ERPF ratio of the lesion-side kidney is linked to postoperative eGFR decline following nephrectomy.

## MATERIALS AND METHODS

### Ethics Statements

The Institutional Review Board at Taipei Veterans General Hospital approved this study (Approval ID: 2020-12-007BC) and waived the need for informed consent to publish innominate data.

### Study Population and Design

**Figure 1:**
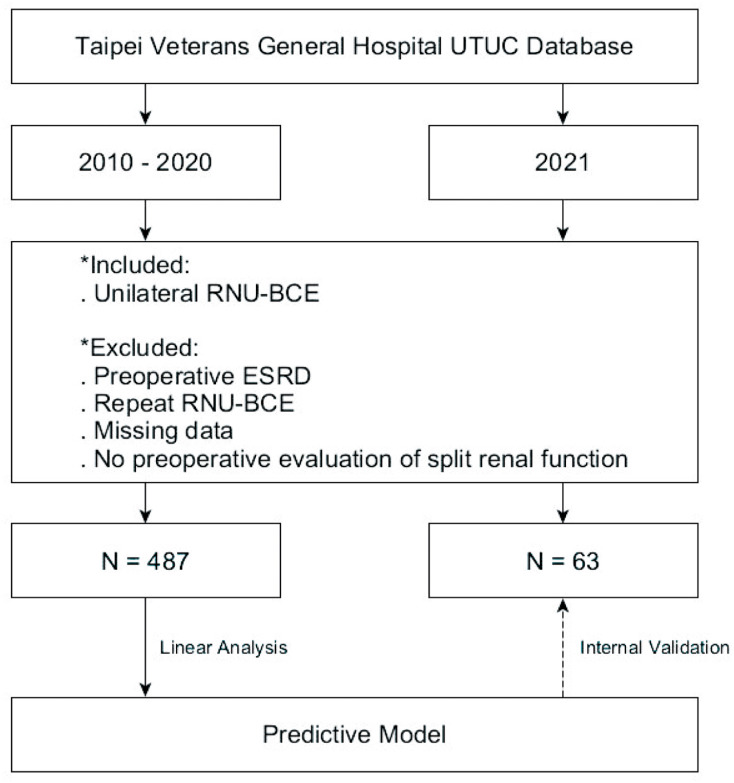
Flow of Patients through the Study.

This retrospective study included patients listed in the UTUC database who were treated by unilateral RNU-BCE at our tertiary referral medical center between January 2010 and December 2020 ( Figure-1 ). The exclusion criteria comprised preoperative end-stage renal disease (ESRD), a second RNU-BCE, missing data, and no preoperative split renal function evaluation data.

ESRD = end-stage renal disease; RNU-BCE = radical nephroureterectomy with bladder cuff excision; UTUC = upper tract urothelial carcinoma.

### Surgical Technique

For minimally invasive RNU-BCE, the procedure was conducted using either a laparoscopic or robot-assisted approach. The RNU was carried out using standard techniques, with the renal pedicle secured using a laparoscopic endovascular stapler. BCE was performed mostly in an extravesical fashion. For open RNU-BCE, the RNU was similarly performed in a standard fashion, with the renal pedicle controlled using either a stapler, or traditional ligation method.

### Data Acquisition

We staged UTUC according to the American Joint Committee on Cancer, 8th edition (2017). Complications were graded on the basis of the Clavien–Dindo Classification system. Serum creatinine was measured at baseline and at 3 and 6 months postoperatively. The eGFR was calculated using the Cockcroft-Gault equation and normalized to a standard body surface area of 1.73 m^2^ ( 9 ).

## ERPF

ERPF was quantified by renography using a gamma camera in supine patients injected intravenously with a bolus of 296 MBq of Technetium-99m mercaptoacetyltriglycine (Tc-99m MAG3). Radiotracer perfusion was visualized using posterior dynamic image acquisition. Time-activity curves were created on the basis of radionuclide activity over regions of interest, that is, both kidneys. Flow into each kidney was compared with that into the aorta to calculate renal perfusion.

### Statistical Analysis

Multivariable linear regression analysis was performed to identify independent predictors of eGFR decline at 3 months postoperatively. Clinical variables included in the multivariable linear regression analysis were selected a priori based on clinical relevance, prior literature, and data availability within our database. Linear relationships were determined, and a predictive equation was developed from scatter plots. All values with P < 0.05 were considered statistically significant. All data were statistically analyzed using IBM SPSS Statistics for Windows, Version 25.0 (IBM Corp., Armonk, NY, USA).

### Internal Validation

We applied the same inclusion criteria to our database and selected patients from 2021 as the target to internally validate our equation ( Figure-1 ). We assessed whether our equation could reasonable predict postoperative eGFR values. Cisplatin ineligibility was defined as eGFR <60 mL/min/1.732 m^2^. RNU-related cisplatin ineligibility was defined as patients who were preoperatively eligible for cisplatin therapy but became ineligible after unilateral RNU owing to insufficient residual renal function.

## RESULTS

Among 694 patients who underwent unilateral RNU-BCE for UTUC at our institute between January 2010 and December 2020, 207 met the exclusion criteria. We analyzed data from 487 eligible patients ( Figure-1 ). Table-1 shows the demographic data. The median age at the time of surgery was 73.0 (interquartile range [IQR], 66.0–80.0) years and the male-to-female ratio was almost 1:1 (49.7:50.3%) ( Supplementary Table-1 ).

**Table 1:** Demographic data of patients with upper tract urothelial carcinoma.

Demographic		All patients (n = 487)
**Sex**		
	Male	242 (49.7%)
	Female	245 (50.3%)
Median age at surgery, y		73.0 (66.0-80.0)
Body mass index, kg/m^2^		24.24 (21.7-26.9)
**ECOG^2^ performance status**		
	< 2	469 (96.3%)
	≥ 2	18 (3.7%)
Hypertension		272 (55.9%)
Diabetes mellitus		143 (29.4%)
Coronary heart disease		71 (14.6%)
Smoker		102(20.9%)
Chinese herbs		13 (2.7%)
Hydronephrosis		277 (56.9%)
**Preoperative bladder cancer**		
	No	399 (81.9%)
	Yes	29 (6.0%)
Concomitant		59 (12.1%)
Neoadjuvant systemic therapy		16 (3.3%)
**Surgical procedure**		
	Open	131 (26.9%)
	Minimally Invasive	356 (73.1%)
	Surgical duration, min.	340 (275-409)
Blood loss, mL		200 (50-350)
Blood transfusion		88 (18.1%)
**Clavien–Dindo classification**		
	1,2	90 (18.5%)
	≥ 3	13 (2.7%)
**Pathology**		
	< pT3	293 (60.2%)
	≥ pT3	194 (39.8%)
	pN (+)	44 (8.6%)
	Multifocality	118 (24.2%)
	Variant histology	38 (7.8%)

Data is shown as n% or medians (IQR). Eastern Cooperative Oncology Group; IQR = interquartile range.

Table-2 shows details of renal function. The median total preoperative ERPF was 241.30 (IQR, 184.49–309.00) mL/min., for which the kidney on the lesion side accounted for 35.60% (IQR, 20.83%–46.41%). At 3 months postoperatively, the median postoperative eGFR decline ratio was 24.03% (IQR, 9.26%–36.25%). We found that 142 (29.2%) of 487 patients were preoperatively eligible for cisplatin-based therapy, and that 48 (10.0%) remained eligible after unilateral RNU at 3 months postoperatively. Thirty (6.2%) patients eventually developed ESRD and required renal replacement therapy after surgery with a median latency of 2.1 (IQR, 0.6–4.4) years. All patients were postoperatively followed up for a median of 2.75 (IQR, 1.2–5.3) years ( Supplementary Table-2 ).

**Table 2:** Renal function profile and split renal function test results.

Renal function profile	
**Preoperative**	**All Patients (n = 487)**
Creatinine, mg/dL	1.06 (0.84–1.42)
eGFR, mL/min/1.732 m^2^	47.27 (33.34–63.12)
Total ERPF, mL/min.	241.30 (184.49–309.00)
Lesion Side/Total ERPF ratio, %	35.60 (20.83–46.41)
Cisplatin eligibility	142 (29.2%)
**Postoperative 3 months**	**N = 487**
Creatinine, mg/dL	1.43 (1.15–1.89)
eGFR, mL/min/1.732 m^2^	35.12 (26.02–47.05)
eGFR decline, %	24.03 (9.26–36.25)
Cisplatin eligibility	48 (10.0%)
**Postoperative 6 months**	**N = 386**
Creatinine, mg/dL	1.43 (1.19–1.86)
eGFR, mL/min/1.732 m^2^	36.10 (25.70–47.01)
eGFR decline, %	26.19 (12.52–37.24)
Cisplatin eligibility	31 (8.0%)
**Dialysis**	**N = 487**
Required after surgery	30 (6.2%)
Postoperative requirement for dialysis, y	2.1 (0.6–4.4)

Data are shown as n% or medians (IQR) unless otherwise stated.

eGFR = estimated glomerular filtration rate; ERPF = effective renal plasma flow; IQR = interquartile range; y = years

**Figure 2:**
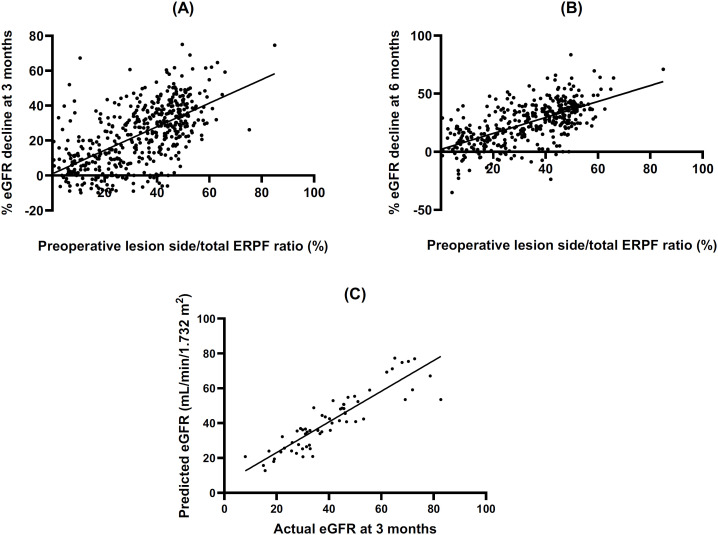
ERPF ratio and postoperative eGFR decline with validation.

Multivariable linear regression analysis identified several significant predictors of eGFR decline at 3 months postoperatively ( Table-3 ). A higher Lesion Side/Total ERPF ratio (%) was significantly associated with greater eGFR decline (B = 0.627, 95% CI: 0.545 to 0.709, β = 0.570, p < 0.001). Scatter plots demonstrated a significant and linear correlation between the preoperative lesion side/total ERPF and the postoperative % eGFR decline. The equation at 3 and 6 months postoperatively predicted eGFR decline (%) = 0.675 x lesion side/total ERPF (%) + 0.919 (correlation coefficient (r) = 0.613, P < 0.001; Figure-2A ) and eGFR decline (%) = 0.643 x lesion side/total ERPF (%) + 4.153 (r = 0.614, P < 0.001; Figure-2B ).

**Table 3:** Multivariable linear regression for predictors of eGFR decline at 3 months postoperatively.

Variables	B (Unstandardized Coefficient)	95% CI for B β	Standardized	p-value
Sex (male vs female)	0.125	-2.689 to 2.939	0.004	0.930
Age at surgery, y	0.239	0.077 to 0.401	0.135	0.004
Body mass index, kg/m^2^	-0.204	-0.563 to 0.156	-0.45	0.266
Hypertension (yes vs no)	-1.286	-3.978 to 1.405	-0.037	0.348
Diabetes mellitus (yes vs no)	-0.897	-3.642 to 1.847	-0.023	0.521
Coronary heart disease (yes vs no)	0.082	-3.644 to 3.808	0.002	0.965
Smoker (yes vs no)	-1.772	-5.165 to 1.622	-0.041	0.306
Chinese herbs (yes vs no)	4.534	-3.020 to 12.089	0.042	0.239
Hydronephrosis (yes vs no)	-2.42	-2.810 to 2.326	-0.007	0.853
Surgical procedure (minimally invasive vs open)	1.879	-0.863 to 4.621	0.048	0.179
Blood loss, mL	-0.001	-0.002 to 0.000	-0.075	0.048
Clavien–Dindo classification (≥ 3 vs 1,2)	7.273	-0.753 to 15.299	0.067	0.076
Preoperative eGFR, mL/min/1.732 m^2^	0.158	0.081 to 0.235	0.204	<0.001
Lesion Side/Total ERPF ratio, %	0.627	0.545 to 0.709	0.0570	<0.001

eGFR = estimated glomerular filtration rate; ERPF = effective renal plasma flow; y = years.

(A) Scatter plot of preoperative lesion side/total ERPF ratio vs. % eGFR decline at 3 months post-operatively. eGFR decline (%) = 0.675 × ERPF ratio (%) + 0.919 (r = 0.613; R^2^ = 0.376; P < 0.001).

(B) Scatter plot of preoperative lesion side/total ERPF ratio vs. % eGFR decline at 6 months post-operatively. eGFR decline (%) = 0.643 × ERPF ratio (%) + 4.153 (r = 0.614; R^2^ = 0.377; P < 0.001).

(C) Internal validation using patient data in 2021, showing predicted vs. actual eGFR at 3 months post-operatively (r = 0.896; R^2^ = 0.803; P < 0.001).

eGFR = estimated glomerular filtration rate; ERPF = effective renal plasma flow.

We internally validated these findings using data from 63 eligible patients in the UTUC database who underwent unilateral RNU in 2021. Our equation validly predicted an eGFR decline at 3 months postoperatively, with positive correlation between the predicted and actual eGFR values (r = 0.896, P < 0.001; Figure-2C ). Twenty-two (34.9%) patients were eligible for cisplatin-based therapy preoperatively, and 10 (15.9%) remained eligible at 3 months after unilateral RNU. Our equation predicted renal function loss with 100% accuracy in all 12 patients who experienced RNU-related cisplatin ineligibility.

## DISCUSSION

A higher Lesion Side/Total ERPF ratio was significantly associated with greater postoperative eGFR decline, indicating that greater functional loss from the affected kidney leads to more pronounced renal impairment. To enhance clinical applicability, we developed predictive models for eGFR decline at 3 and 6 months postoperatively using the Lesion Side/Total ERPF ratio as the sole predictor. This approach was intentionally simplified to create a practical tool that can be easily implemented in routine clinical decision-making without the need for complex multivariable inputs. Internal validation confirmed the equation reliably predicted eGFR decline and identified patient's ineligible for postoperative cisplatin.

The literature shows that several models and nomograms for post-nephrectomy renal function prediction have been developed ( 10 - 14 ). Predictive models for post-nephrectomy renal function have been based on age and weight ( 11 ), and on age, diabetes, preoperative eGFR and proteinuria, tumor size, elapsed time after surgery, and interaction between elapsed time after surgery and age ( 14 ).

The eGFR estimates the amount of urine filtered by both kidneys per minute, whereas ERPF detects the fractional amount of plasma flowing through each kidney. Although derived from different mechanisms, these values correlate ( 15 ). The potential predictive value of ERPF for postoperative residual renal function has been assessed using Tc-99m MAG3 ( 5 ). Preoperative MAG3 clearance in the remaining kidney moderately correlated with postoperative creatinine clearance (n = 35, r = 0.596; P = 0.0005), and preoperative MAG3 clearance of 130 mL/min. in the remaining kidney was established as a cutoff for postoperative renal insufficiency. A predictive equation for eGFR at 1 year post-RNU has been proposed, based on preoperative MAG3 renal scintigraphy results ( 6 ). The preoperative ERPF of the resected kidney moderately correlated with a postoperative eGFR decrease (n = 129, r = 0.528; P < 0.001). However, a convincing predictive equation was not established owing to small samples. Furthermore, a cutoff value rather than the ratio of postoperative ERPF of the resected kidney was used to predict postoperative eGFR, whereas we believe that the ratio for predicting postoperative eGFR is a better approach.

Computed tomography (CT) volumetry has been widely used to assess split kidney function in kidney donors, and may be the preferred modality over technetium-99m (Tc-99m) diethylenetriamine penta-acetic acid (DTPA) nuclear renography ( 16 ). However, inaccuracy of residual renal function prediction by parenchymal volume analysis was independently associated with pyelonephritis, hydronephrosis, renal vein thrombosis, and infiltrative features ( 17 ). Low contralateral kidney volume (< 150 mL) was associated with new-onset chronic kidney disease in patients with advanced upper tract urothelial cancer ( 18 ). CT renal cortex radiodensity for the intact kidney is considered a useful tool for predicting unremoved kidney function in UTUC patients after comparing with DTPA nuclear renography ( 7 ). The utility of CT volumetry for UTUC patient post-RNU renal function prediction warrants further validation.

Our institutional policy of routine MAG3 renography before RNU aims to better assess preoperative renal function, particularly in patients with comorbidities or marginal renal reserve. Various radiotracers are used in renal scintigraphy; Tc-99m MAG3 is the standard for renogram imaging, while Tc-99m DTPA is used solely for GFR estimation. MAG3 is preferred for patients with impaired renal function or suspected obstruction due to its higher extraction fraction (40-50%) compared to DTPA (20%), as outlined in the SNMMI/EANM guidelines for diuretic renal scintigraphy ( 19 , 20 ). Given that nearly 60% of patients presented with hydronephrosis, our institution selected the MAG3 ERPF method as the preferred radiotracer. Both ERPF with MAG3 and GFR with DTPA are covered under Taiwan's National Health Insurance, enabling access to comprehensive renal function evaluation without additional cost ( 21 ). The normal ERPF for individuals aged 61–80 is 511.3 ± 91.2, with a typical waiting period of 1–3 weeks for the scan at our institute ( 22 ).

Postoperative eGFR has recently been predicted by Tc-99m DTPA renal dynamic images of 1,286 patients with a single renal mass that was treated by radical or partial nephrectomy ( 8 ). On the basis of finding a good correlation (R2 = 0.554), the authors proposed using a multivariate predictive model. However, we focused on patients with UTUC that were treated by unilateral nephroureterectomy. Thus, our equation could potentially be applied to patients requiring neoadjuvant chemotherapy before surgery. The concepts in these two studies are completely different.

Unlike 15%–25% of bladder urothelial carcinomas, 60% of UTUCs are already invasive at initial diagnosis ( 23 ). Therefore, surgery followed by surveillance is insufficient for patients with locally advanced UTUC. Adjuvant chemotherapy with a cisplatin-based regimen improves disease-free survival in patients with locally advanced staging ( 24 ). However, the post-RNU loss of renal function might render such patient's ineligible for adjuvant chemotherapy. Renal function eligibility according to the Galsky criterion for cisplatin-based chemotherapy is generally eGFR ≥ 60 mL/min/1.732 m^2^ ( 25 ). Although there is less evidence compared with adjuvant therapy ( 26 ), preoperative chemotherapy might be considered if post-RNU cisplatin ineligibility is likely. A judicious treatment plan can be created if predictions of postoperative renal function are reliable.

Postoperative renal insufficiency is not defined using an established cutoff value. However, post-RNU cisplatin eligibility is a key concern when locally advanced disease is pathologically confirmed. Thus, we tested the accuracy of our equation for predicting cisplatin eligibility after RNU using internal validation. Our model was promising, indicating that the predictive equation can help to preoperatively optimize treatment strategies.

The low incidence of postoperative dialysis likely reflects careful planning and case selection. The 5-year overall survival rate of patients receiving renal replacement therapy was 49.1% in the most recent cohort ( 27 ), which was just as lethal as the cancer itself. Surgery is most likely to become a concern when dialysis is inevitable in the future.

Our study has several limitations. Our analysis used a single-center, retrospective design, which may have introduced bias due to limited population diversity. However, the homogeneity of this single-center study, characterized by similar practice patterns and follow-up protocols, strengthens the credibility of the results. Our predictive model was created using only the Cockcroft-Gault equation and not the Modification of Diet in Renal Disease (MDRD) equation for creatinine clearance estimation, which is another popular equation for renal function assessment. Our UTUC database is based on an Asian population, and the MDRD equation is less accurate in Asians because the equation originated from a cohort of Caucasians and Africans ( 28 - 30 ). Nevertheless, short-term changes of body weight such as edema or dehydration might have compromised the accuracy of the calculation.

Renal function can be influenced by many factors; thus, we evaluated postoperative changes in renal function at 3 and 6 months to avoid immediate influences of factors associated with surgery, such as surgical approach, blood loss, postoperative complications, volume depletion or analgesic use. We did not evaluate long-term changes to avoid many variables that might affect renal function aside from unilateral nephrectomy. Postoperative renal function evaluated at 3 and 6 months was also crucial to assessing whether patients were eligible for adjuvant therapy. We did not include additional demographic factors in developing the multivariate nomographic model. Given the short time frame following RNU, it is reasonable to assume that individual demographic characteristics remain stable before and after surgery. Therefore, we based our predictive equation on the most authoritative single factor, presenting it as a simple linear formula to offer a practical and accessible predictive tool.

While the accessibility of renal scintigraphy and radiotracers varies between centers and countries, our predictive model based on the MAG3 ERPF test still offers valuable insights, with potential considerations for broader application and clinical translation. Despite internal validation, the tested group was smaller than of the original group that generated the predictive equation. Prospective studies of larger cohorts at other institutions are warranted to validate our equation.

## CONCLUSIONS

We developed a predictive equation based on preoperative ERPF data to predict changes in eGFR at 3 and 6 months postoperatively. This equation can predict residual renal function and RNU-related cisplatin eligibility before unilateral RNU. However, further external validation is required to confirm our findings.

## Data Availability

The data sets generated during and/or analyzed during the current study are available from the corresponding author on reasonable request.

## ABBREVIATIONS

UTUC: urinary tract urothelial carcinoma
RNU: radical nephroureterectomy
RNU-BCE: Radical nephroureterectomy with bladder cuff excision
ESRD: end-stage renal disease
eGFR: estimated glomerular filtration rate
ERPF: effective renal plasma flow
Tc-99m MAG3: 296 MBq of Technetium-99m mercaptoacetyltriglycine
IQR: interquartile range
MDRD: Modification of Diet in Renal Disease
