# Supplementary material for: Equation Predicts Renal Function after Nephroureterectomy to Treat Upper Tract Urothelial Carcinoma
Source: Int Braz J Urol. 2025 Sep 30;52(1):e20250076. doi: 10.1590/S1677-5538.IBJU.2025.0076 (PMC12974961; doi:10.1590/S1677-5538.IBJU.2025.0076)
Supplement: Supplementary file 1 [file 1677-6119-ibju-52-01-e20250076-Suppl1.pdf]

APPENDIX:

Supplement Table 1 - Detail demographic data of patients with upper tract urothelial carcinoma.

All patients (n = 487)	N (%)	Mean ± SD	Median (IQR)	Min – Max
**Sex**				
Male	242 (49.7%)			
Female	245 (50.3%)			
Age at surgery, y		72.8 ± 9.9	73.0 (66.0–80.0)	21 - 95
Body mass index, kg/m^2		24.51 ± 3.88	24.24 (21.7–26.9)	15.05 – 39.97
**ECOG[2] performance status**				
< 2	469 (96.3%)			
≥ 2	18 (3.7%)			
Hypertension	272 (55.9%)			
Diabetes mellitus	143 (29.4%)			
Coronary heart disease	71 (14.6%)			
Smoker	102(20.9%)			
Chinese herbs	13 (2.7%)			
Hydronephrosis	277 (56.9%)			
**Preoperative bladder cancer**				
No	399 (81.9%)			
Yes	29 (6.0%)			
Concomitant	59 (12.1%)			
Neoadjuvant systemic therapy	16 (3.3%)			
**Surgical procedure**				
Open	131 (26.9%)			
Minimally Invasive	356 (73.1%)			
Surgical duration, min		354.6 ± 116.0	340 (275–409)	145 - 1200
Blood loss, mL		389.9 ± 1248.6	200 (50–350)	30 - 18800
Blood transfusion	88 (18.1%)			
**Clavien-Dindo classification**				
1,2	90 (18.5%)			
≥ 3	13 (2.7%)			
**Pathology**				
< pT3	293 (60.2%)			
≥ pT3	194 (39.8%)			
pN (+)	44 (8.6%)			
Multifocality	118 (24.2%)			
Variant histology	38 (7.8%)			

Eastern Cooperative Oncology Group; IQR = interquartile range; SD = standard deviation.

Supplement Table 2 - Detail renal function profile and split renal function test results.

Renal function profile			
**Preoperative (N = 487)**	**N (%)**	**Mean ± SD**	**Median (IQR)**
Creatinine, mg/dL		1.25 ± 0.69	1.06 (0.84–1.42)
eGFR, mL/min/1.732 m^2		50.56 ± 22.54	47.27 (33.34–63.12)
Total ERPF, mL/min.		252.91 ± 98.64	241.30 (184.49–309.00)
Lesion Side/Total ERPF ratio, %		33.22 ± 15.88	35.60 (20.83–46.41)
Cisplatin eligibility	142 (29.2%)		
**Postoperative 3 months (N = 487)**			
Creatinine, mg/dL		1.70 ± 1.10	1.43 (1.15–1.89)
eGFR, mL/min/1.732 m^2		37.74 ± 16.54	35.12 (26.02–47.05)
eGFR decline, %		23.34 ± 17.47	24.03 (9.26–36.25)
Cisplatin eligibility	48 (10.0%)		
**Postoperative 6 months (N = 386)**			
Creatinine, mg/dL		1.75 ± 1.21	1.43 (1.19–1.86)
eGFR, mL/min/1.732 m^2		37.59 ± 16.34	36.10 (25.70–47.01)
eGFR decline, %		24.84 ± 17.59	26.19 (12.52–37.24)
Cisplatin eligibility	31 (8.0%)		
**Dialysis (N = 487)**			
Required after surgery	30 (6.2%)		
Postoperative requirement for dialysis, y		3.0 ± 3.48	2.1 (0.6–4.4)

eGFR = estimated glomerular filtration rate; ERPF = effective renal plasma flow; SD = standard deviation; IQR = interquartile range; y = years.
